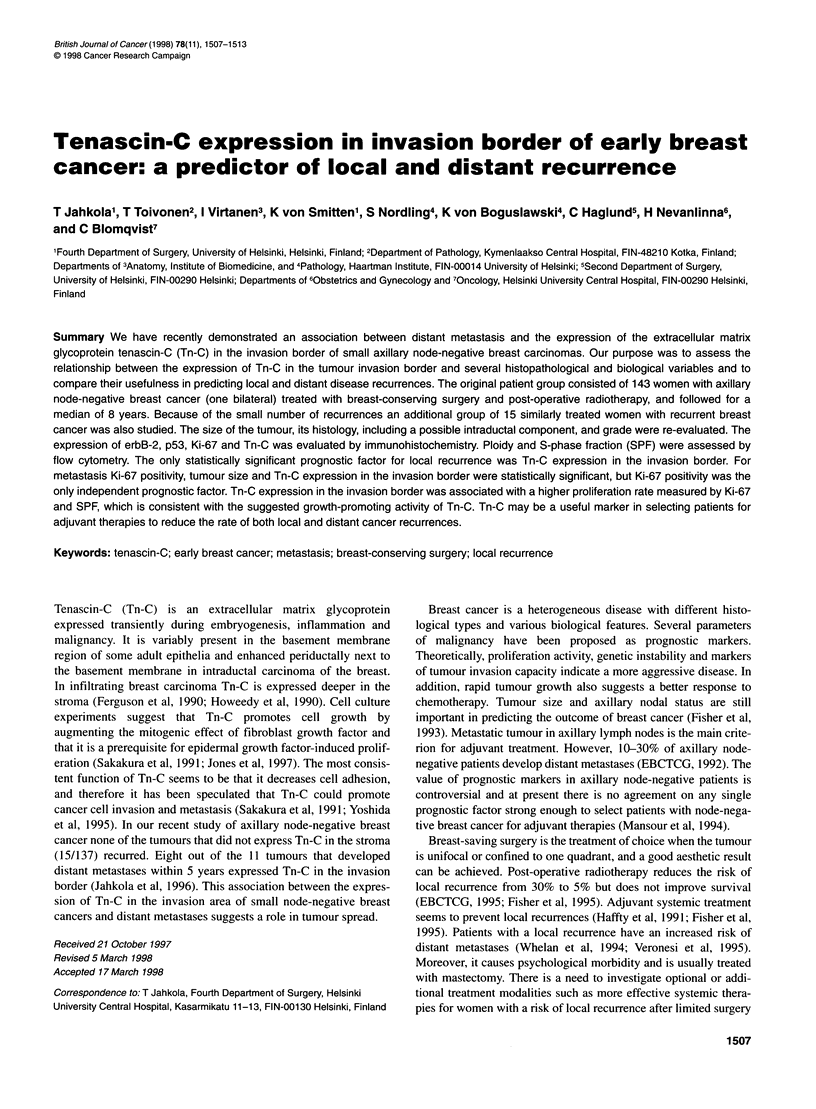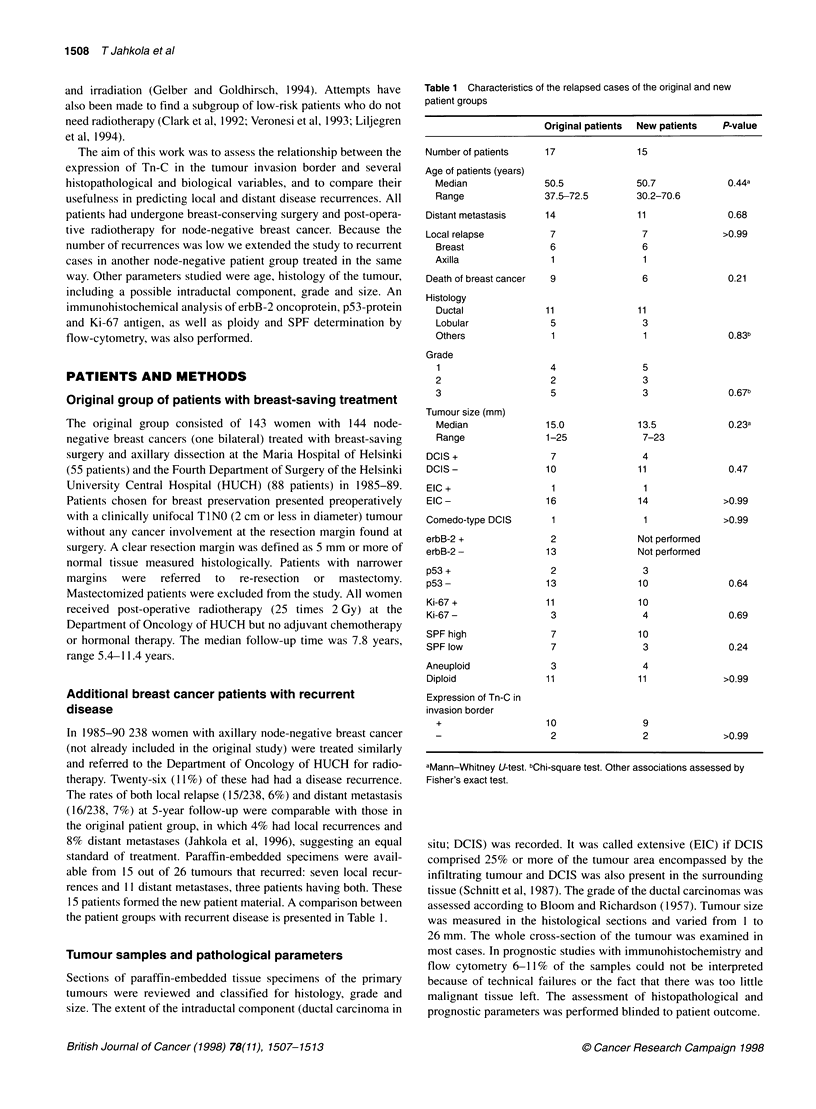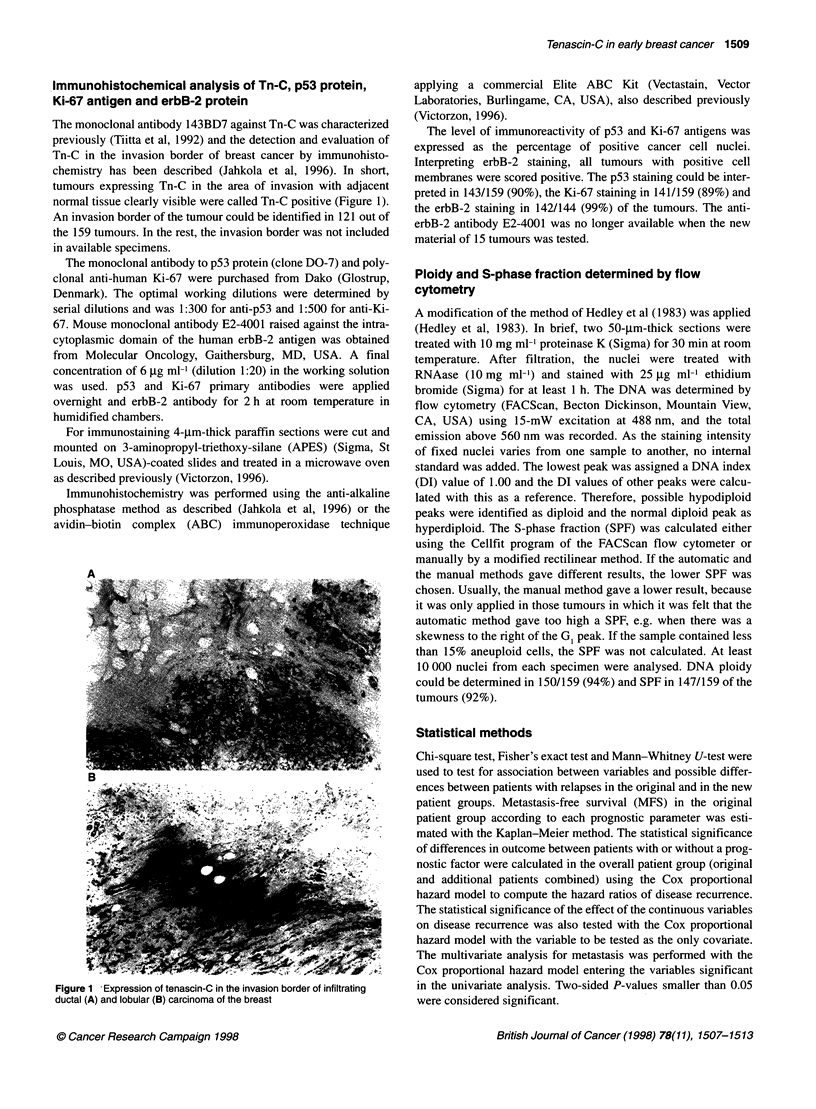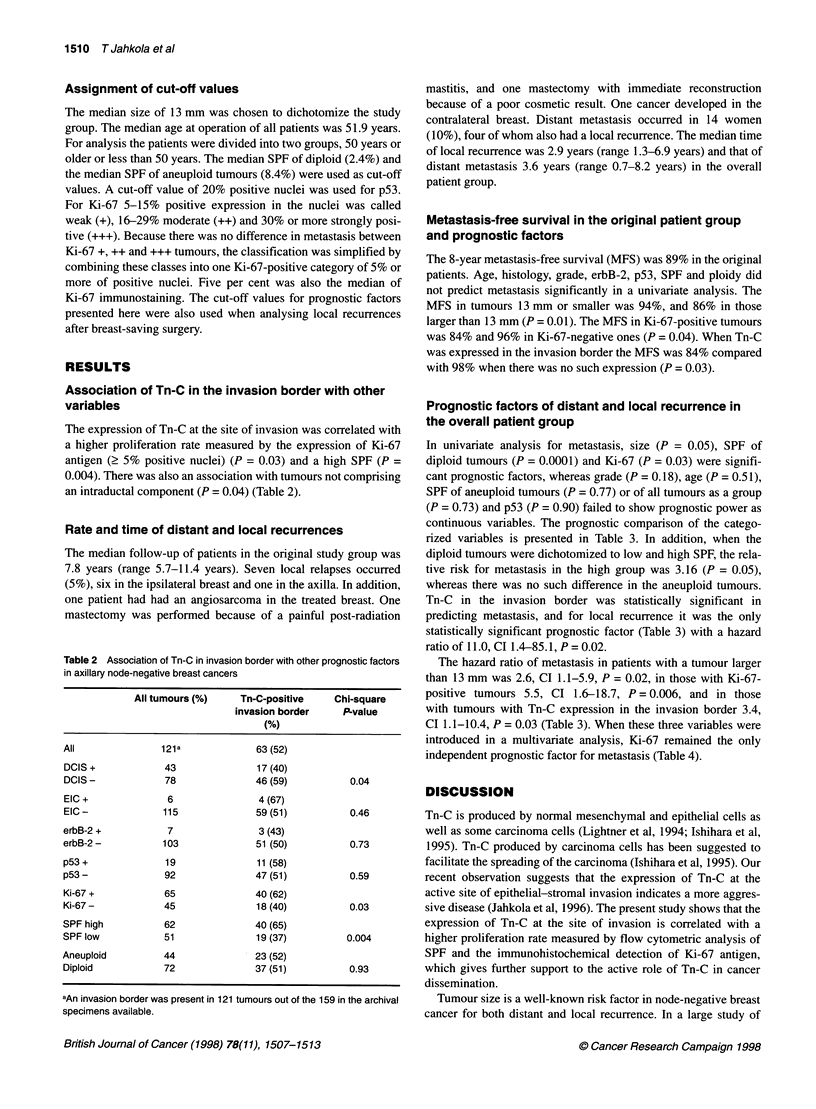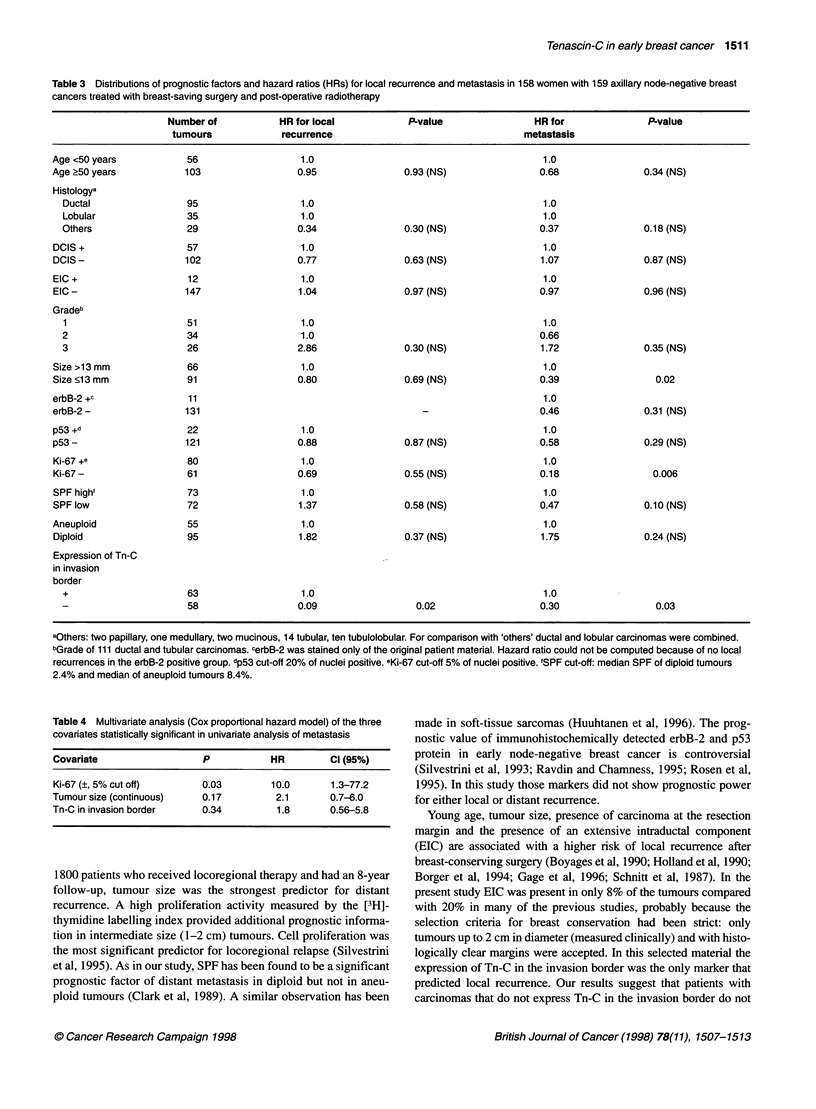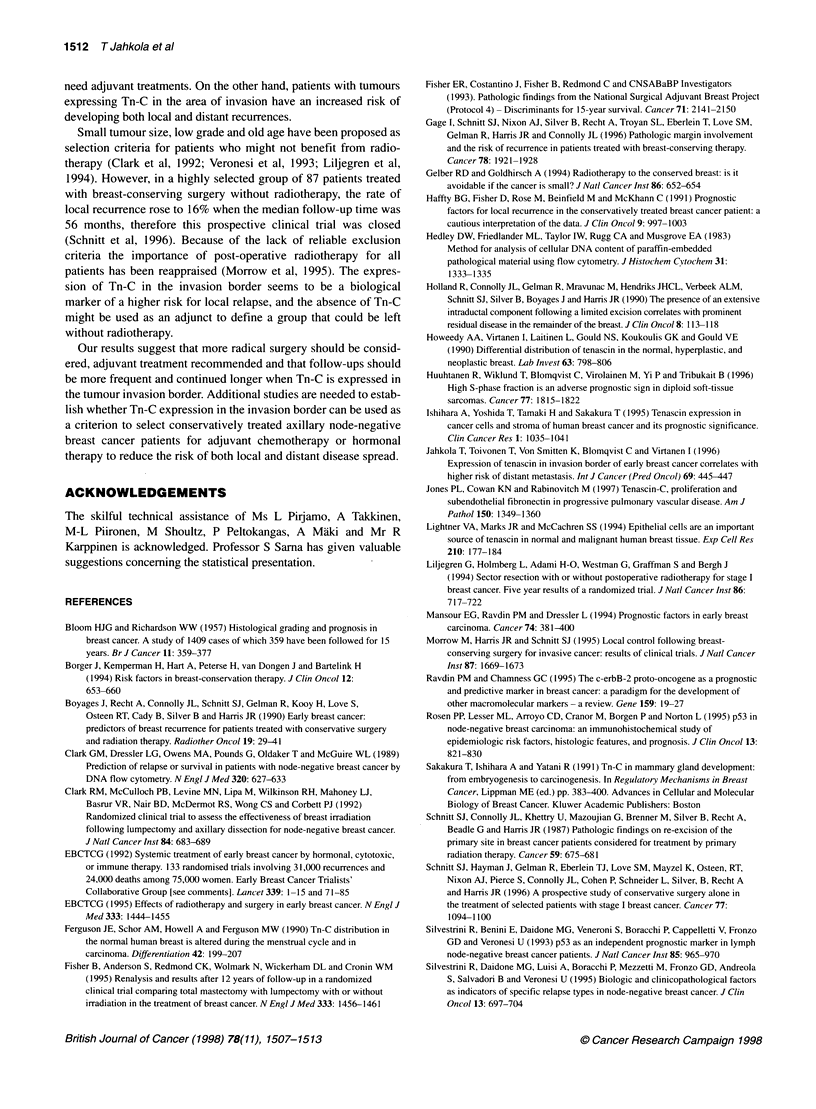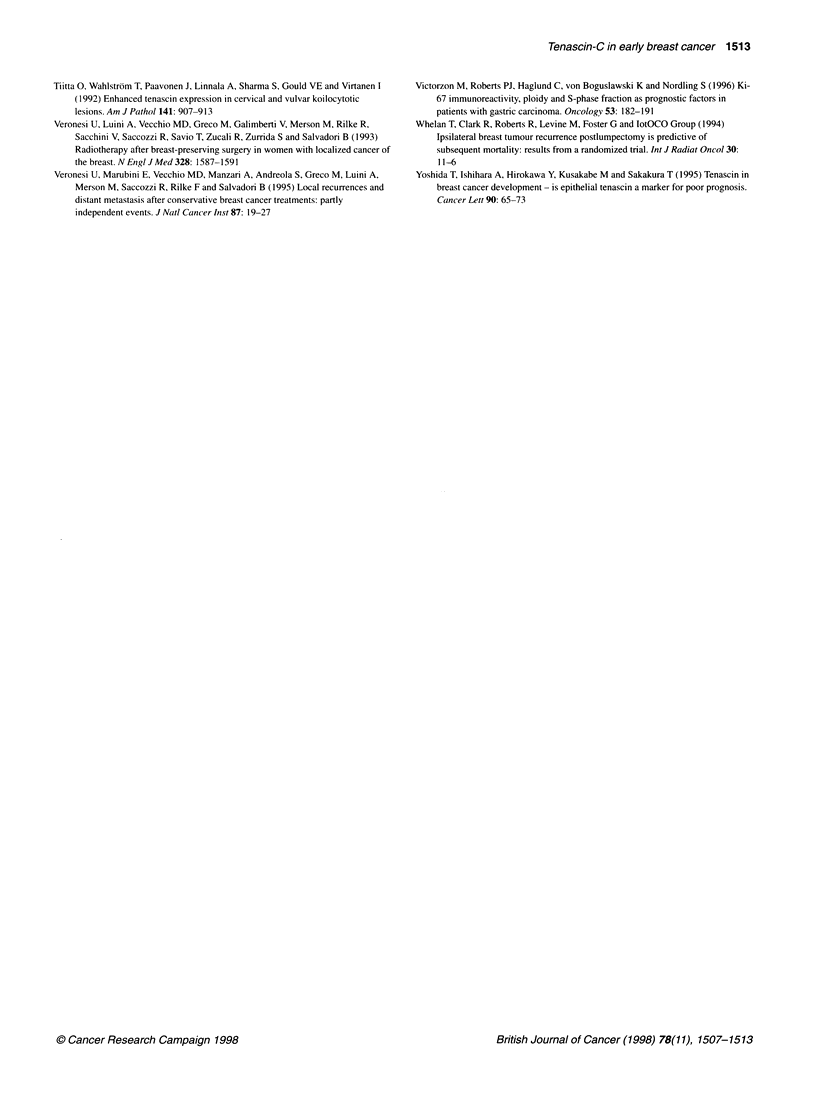# Tenascin-C expression in invasion border of early breast cancer: a predictor of local and distant recurrence.

**DOI:** 10.1038/bjc.1998.714

**Published:** 1998-12

**Authors:** T. Jahkola, T. Toivonen, I. Virtanen, K. von Smitten, S. Nordling, K. von Boguslawski, C. Haglund, H. Nevanlinna, C. Blomqvist

**Affiliations:** Fourth Department of Surgery, University of Helsinki, Finland.

## Abstract

**Images:**


British Journal of Cancer (1998) **78**(11), 1507–1513
© 1998 Cancer Research Campaign

### Tenascin-C expression in invasion border of early breast cancer: a predictor of local and distant recurrence

**T Jahkola[1], T Toivonen[2], I Virtanen[3], K von Smitten[1], S Nordling[4], K von Boguslawski[4], C Haglund[5], H Nevanlinna[6], and C Blomqvist[7]**

[1]Fourth Department of Surgery, University of Helsinki, Helsinki, Finland; [2]Department of Pathology, Kymenlaakso Central Hospital, FIN-48210 Kotka, Finland; Departments of [3]Anatomy, Institute of Biomedicine, and [4]Pathology, Haartman Institute, FIN-00014 University of Helsinki; [5]Second Department of Surgery, University of Helsinki, FIN-00290 Helsinki; Departments of [6]Obstetrics and Gynecology and [7]Oncology, Helsinki University Central Hospital, FIN-00290 Helsinki, Finland

**Summary** We have recently demonstrated an association between distant metastasis and the expression of the extracellular matrix glycoprotein tenascin-C (Tn-C) in the invasion border of small axillary node-negative breast carcinomas. Our purpose was to assess the relationship between the expression of Tn-C in the tumour invasion border and several histopathological and biological variables and to compare their usefulness in predicting local and distant disease recurrences. The original patient group consisted of 143 women with axillary node-negative breast cancer (one bilateral) treated with breast-conserving surgery and post-operative radiotherapy, and followed for a median of 8 years. Because of the small number of recurrences an additional group of 15 similarly treated women with recurrent breast cancer was also studied. The size of the tumour, its histology, including a possible intraductal component, and grade were re-evaluated. The expression of erbB-2, p53, Ki-67 and Tn-C was evaluated by immunohistochemistry. Ploidy and S-phase fraction (SPF) were assessed by flow cytometry. The only statistically significant prognostic factor for local recurrence was Tn-C expression in the invasion border. For metastasis Ki-67 positivity, tumour size and Tn-C expression in the invasion border were statistically significant, but Ki-67 positivity was the only independent prognostic factor. Tn-C expression in the invasion border was associated with a higher proliferation rate measured by Ki-67 and SPF, which is consistent with the suggested growth-promoting activity of Tn-C. Tn-C may be a useful marker in selecting patients for adjuvant therapies to reduce the rate of both local and distant cancer recurrences.

**Keywords:** tenascin-C; early breast cancer; metastasis; breast-conserving surgery; local recurrence

Tenascin-C (Tn-C) is an extracellular matrix glycoprotein expressed transiently during embryogenesis, inflammation and malignancy. It is variably present in the basement membrane region of some adult epithelia and enhanced periductally next to the basement membrane in intraductal carcinoma of the breast. In infiltrating breast carcinoma Tn-C is expressed deeper in the stroma (Ferguson et al, 1990; Howeedy et al, 1990). Cell culture experiments suggest that Tn-C promotes cell growth by augmenting the mitogenic effect of fibroblast growth factor and that it is a prerequisite for epidermal growth factor-induced proliferation (Sakakura et al, 1991; Jones et al, 1997). The most consistent function of Tn-C seems to be that it decreases cell adhesion, and therefore it has been speculated that Tn-C could promote cancer cell invasion and metastasis (Sakakura et al, 1991; Yoshida et al, 1995). In our recent study of axillary node-negative breast cancer none of the tumours that did not express Tn-C in the stroma (15/137) recurred. Eight out of the 11 tumours that developed distant metastases within 5 years expressed Tn-C in the invasion border (Jahkola et al, 1996). This association between the expression of Tn-C in the invasion area of small node-negative breast cancers and distant metastases suggests a role in tumour spread.

*Received 21 October 1997*
*Revised 5 March 1998*
*Accepted 17 March 1998*

*Correspondence to:* T Jahkola, Fourth Department of Surgery, Helsinki University Central Hospital, Kasarmikatu 11–13, FIN-00130 Helsinki, Finland

Breast cancer is a heterogeneous disease with different histological types and various biological features. Several parameters of malignancy have been proposed as prognostic markers. Theoretically, proliferation activity, genetic instability and markers of tumour invasion capacity indicate a more aggressive disease. In addition, rapid tumour growth also suggests a better response to chemotherapy. Tumour size and axillary nodal status are still important in predicting the outcome of breast cancer (Fisher et al, 1993). Metastatic tumour in axillary lymph nodes is the main criterion for adjuvant treatment. However, 10–30% of axillary node-negative patients develop distant metastases (EBCTCG, 1992). The value of prognostic markers in axillary node-negative patients is controversial and at present there is no agreement on any single prognostic factor strong enough to select patients with node-negative breast cancer for adjuvant therapies (Mansour et al, 1994).

Breast-saving surgery is the treatment of choice when the tumour is unifocal or confined to one quadrant, and a good aesthetic result can be achieved. Post-operative radiotherapy reduces the risk of local recurrence from 30% to 5% but does not improve survival (EBCTCG, 1995; Fisher et al, 1995). Adjuvant systemic treatment seems to prevent local recurrences (Haffty et al, 1991; Fisher et al, 1995). Patients with a local recurrence have an increased risk of distant metastases (Whelan et al, 1994; Veronesi et al, 1995). Moreover, it causes psychological morbidity and is usually treated with mastectomy. There is a need to investigate optional or additional treatment modalities such as more effective systemic therapies for women with a risk of local recurrence after limited surgery and irradiation (Gelber and Goldhirsch, 1994). Attempts have also been made to find a subgroup of low-risk patients who do not need radiotherapy (Clark et al, 1992; Veronesi et al, 1993; Liljegren et al, 1994).

The aim of this work was to assess the relationship between the expression of Tn-C in the tumour invasion border and several histopathological and biological variables, and to compare their usefulness in predicting local and distant disease recurrences. All patients had undergone breast-conserving surgery and post-operative radiotherapy for node-negative breast cancer. Because the number of recurrences was low we extended the study to recurrent cases in another node-negative patient group treated in the same way. Other parameters studied were age, histology of the tumour, including a possible intraductal component, grade and size. An immunohistochemical analysis of erbB-2 oncoprotein, p53-protein and Ki-67 antigen, as well as ploidy and SPF determination by flow-cytometry, was also performed.

### PATIENTS AND METHODS

### Original group of patients with breast-saving treatment

The original group consisted of 143 women with 144 node-negative breast cancers (one bilateral) treated with breast-saving surgery and axillary dissection at the Maria Hospital of Helsinki (55 patients) and the Fourth Department of Surgery of the Helsinki University Central Hospital (HUCH) (88 patients) in 1985–89. Patients chosen for breast preservation presented preoperatively with a clinically unifocal T1N0 (2 cm or less in diameter) tumour without any cancer involvement at the resection margin found at surgery. A clear resection margin was defined as 5 mm or more of normal tissue measured histologically. Patients with narrower margins were referred to re-resection or mastectomy. Mastectomized patients were excluded from the study. All women received post-operative radiotherapy (25 times 2 Gy) at the Department of Oncology of HUCH but no adjuvant chemotherapy or hormonal therapy. The median follow-up time was 7.8 years, range 5.4–11.4 years.

### Additional breast cancer patients with recurrent disease

In 1985–90 238 women with axillary node-negative breast cancer (not already included in the original study) were treated similarly and referred to the Department of Oncology of HUCH for radiotherapy. Twenty-six (11%) of these had had a disease recurrence. The rates of both local relapse (15/238, 6%) and distant metastasis (16/238, 7%) at 5-year follow-up were comparable with those in the original patient group, in which 4% had local recurrences and 8% distant metastases (Jahkola et al, 1996), suggesting an equal standard of treatment. Paraffin-embedded specimens were available from 15 out of 26 tumours that recurred: seven local recurrences and 11 distant metastases, three patients having both. These 15 patients formed the new patient material. A comparison between the patient groups with recurrent disease is presented in Table 1.

### Tumour samples and pathological parameters

Sections of paraffin-embedded tissue specimens of the primary tumours were reviewed and classified for histology, grade and size. The extent of the intraductal component (ductal carcinoma in situ; DCIS) was recorded. It was called extensive (EIC) if DCIS comprised 25% or more of the tumour area encompassed by the infiltrating tumour and DCIS was also present in the surrounding tissue (Schnitt et al, 1987). The grade of the ductal carcinomas was assessed according to Bloom and Richardson (1957). Tumour size was measured in the histological sections and varied from 1 to 26 mm. The whole cross-section of the tumour was examined in most cases. In prognostic studies with immunohistochemistry and flow cytometry 6–11% of the samples could not be interpreted because of technical failures or the fact that there was too little malignant tissue left. The assessment of histopathological and prognostic parameters was performed blinded to patient outcome.

**Table 1** Characteristics of the relapsed cases of the original and new patient groups

| | Original patients | New patients | *P*-value |
|---|---|---|---|
| Number of patients | 17 | 15 | |
| Age of patients (years) | | | |
| Median | 50.5 | 50.7 | 0.44[a] |
| Range | 37.5–72.5 | 30.2–70.6 | |
| Distant metastasis | 14 | 11 | 0.68 |
| Local relapse | 7 | 7 | >0.99 |
| Breast | 6 | 6 | |
| Axilla | 1 | 1 | |
| Death of breast cancer | 9 | 6 | 0.21 |
| Histology | | | |
| Ductal | 11 | 11 | |
| Lobular | 5 | 3 | |
| Others | 1 | 1 | 0.83[b] |
| Grade | | | |
| 1 | 4 | 5 | |
| 2 | 2 | 3 | |
| 3 | 5 | 3 | 0.67[b] |
| Tumour size (mm) | | | |
| Median | 15.0 | 13.5 | 0.23[a] |
| Range | 1–25 | 7–23 | |
| DCIS + | 7 | 4 | |
| DCIS – | 10 | 11 | 0.47 |
| EIC + | 1 | 1 | |
| EIC – | 16 | 14 | >0.99 |
| Comedo-type DCIS | 1 | 1 | >0.99 |
| erbB-2 + | 2 | Not performed | |
| erbB-2 – | 13 | Not performed | |
| p53 + | 2 | 3 | |
| p53 – | 13 | 10 | 0.64 |
| Ki-67 + | 11 | 10 | |
| Ki-67 – | 3 | 4 | 0.69 |
| SPF high | 7 | 10 | |
| SPF low | 7 | 3 | 0.24 |
| Aneuploid | 3 | 4 | |
| Diploid | 11 | 11 | >0.99 |
| Expression of Tn-C in invasion border | | | |
| + | 10 | 9 | |
| – | 2 | 2 | >0.99 |

[a]Mann–Whitney *U*-test. [b]Chi-square test. Other associations assessed by Fisher's exact test.

 © Cancer Research Campaign 1998

### Immunohistochemical analysis of Tn-C, p53 protein, Ki-67 antigen and erbB-2 protein

The monoclonal antibody 143BD7 against Tn-C was characterized previously (Tiitta et al, 1992) and the detection and evaluation of Tn-C in the invasion border of breast cancer by immunohistochemistry has been described (Jahkola et al, 1996). In short, tumours expressing Tn-C in the area of invasion with adjacent normal tissue clearly visible were called Tn-C positive (Figure 1). An invasion border of the tumour could be identified in 121 out of the 159 tumours. In the rest, the invasion border was not included in available specimens.

The monoclonal antibody to p53 protein (clone DO-7) and polyclonal anti-human Ki-67 were purchased from Dako (Glostrup, Denmark). The optimal working dilutions were determined by serial dilutions and was 1:300 for anti-p53 and 1:500 for anti-Ki-67. Mouse monoclonal antibody E2-4001 raised against the intracytoplasmic domain of the human erbB-2 antigen was obtained from Molecular Oncology, Gaithersburg, MD, USA. A final concentration of 6 μg $ml^{-1}$ (dilution 1:20) in the working solution was used. p53 and Ki-67 primary antibodies were applied overnight and erbB-2 antibody for 2 h at room temperature in humidified chambers.

For immunostaining 4-μm-thick paraffin sections were cut and mounted on 3-aminopropyl-triethoxy-silane (APES) (Sigma, St Louis, MO, USA)-coated slides and treated in a microwave oven as described previously (Victorzon, 1996).

Immunohistochemistry was performed using the anti-alkaline phosphatase method as described (Jahkola et al, 1996) or the avidin–biotin complex (ABC) immunoperoxidase technique applying a commercial Elite ABC Kit (Vectastain, Vector Laboratories, Burlingame, CA, USA), also described previously (Victorzon, 1996).

The level of immunoreactivity of p53 and Ki-67 antigens was expressed as the percentage of positive cancer cell nuclei. Interpreting erbB-2 staining, all tumours with positive cell membranes were scored positive. The p53 staining could be interpreted in 143/159 (90%), the Ki-67 staining in 141/159 (89%) and the erbB-2 staining in 142/144 (99%) of the tumours. The anti-erbB-2 antibody E2-4001 was no longer available when the new material of 15 tumours was tested.

A

B

**Figure 1** Expression of tenascin-C in the invasion border of infiltrating ductal (**A**) and lobular (**B**) carcinoma of the breast

### Ploidy and S-phase fraction determined by flow cytometry

A modification of the method of Hedley et al (1983) was applied (Hedley et al, 1983). In brief, two 50-μm-thick sections were treated with 10 mg $ml^{-1}$ proteinase K (Sigma) for 30 min at room temperature. After filtration, the nuclei were treated with RNAase (10 mg $ml^{-1}$) and stained with 25 μg $ml^{-1}$ ethidium bromide (Sigma) for at least 1 h. The DNA was determined by flow cytometry (FACScan, Becton Dickinson, Mountain View, CA, USA) using 15-mW excitation at 488 nm, and the total emission above 560 nm was recorded. As the staining intensity of fixed nuclei varies from one sample to another, no internal standard was added. The lowest peak was assigned a DNA index (DI) value of 1.00 and the DI values of other peaks were calculated with this as a reference. Therefore, possible hypodiploid peaks were identified as diploid and the normal diploid peak as hyperdiploid. The S-phase fraction (SPF) was calculated either using the Cellfit program of the FACScan flow cytometer or manually by a modified rectilinear method. If the automatic and the manual methods gave different results, the lower SPF was chosen. Usually, the manual method gave a lower result, because it was only applied in those tumours in which it was felt that the automatic method gave too high a SPF, e.g. when there was a skewness to the right of the $G_1$ peak. If the sample contained less than 15% aneuploid cells, the SPF was not calculated. At least 10 000 nuclei from each specimen were analysed. DNA ploidy could be determined in 150/159 (94%) and SPF in 147/159 of the tumours (92%).

### Statistical methods

Chi-square test, Fisher's exact test and Mann–Whitney *U*-test were used to test for association between variables and possible differences between patients with relapses in the original and in the new patient groups. Metastasis-free survival (MFS) in the original patient group according to each prognostic parameter was estimated with the Kaplan–Meier method. The statistical significance of differences in outcome between patients with or without a prognostic factor were calculated in the overall patient group (original and additional patients combined) using the Cox proportional hazard model to compute the hazard ratios of disease recurrence. The statistical significance of the effect of the continuous variables on disease recurrence was also tested with the Cox proportional hazard model with the variable to be tested as the only covariate. The multivariate analysis for metastasis was performed with the Cox proportional hazard model entering the variables significant in the univariate analysis. Two-sided *P*-values smaller than 0.05 were considered significant.

© Cancer Research Campaign 1998

### Assignment of cut-off values

The median size of 13 mm was chosen to dichotomize the study group. The median age at operation of all patients was 51.9 years. For analysis the patients were divided into two groups, 50 years or older or less than 50 years. The median SPF of diploid (2.4%) and the median SPF of aneuploid tumours (8.4%) were used as cut-off values. A cut-off value of 20% positive nuclei was used for p53. For Ki-67 5–15% positive expression in the nuclei was called weak (+), 16–29% moderate (++) and 30% or more strongly positive (+++). Because there was no difference in metastasis between Ki-67 +, ++ and +++ tumours, the classification was simplified by combining these classes into one Ki-67-positive category of 5% or more of positive nuclei. Five per cent was also the median of Ki-67 immunostaining. The cut-off values for prognostic factors presented here were also used when analysing local recurrences after breast-saving surgery.

### RESULTS

### Association of Tn-C in the invasion border with other variables

The expression of Tn-C at the site of invasion was correlated with a higher proliferation rate measured by the expression of Ki-67 antigen (≥ 5% positive nuclei) ($P$ = 0.03) and a high SPF ($P$ = 0.004). There was also an association with tumours not comprising an intraductal component ($P$ = 0.04) (Table 2).

### Rate and time of distant and local recurrences

The median follow-up of patients in the original study group was 7.8 years (range 5.7–11.4 years). Seven local relapses occurred (5%), six in the ipsilateral breast and one in the axilla. In addition, one patient had had an angiosarcoma in the treated breast. One mastectomy was performed because of a painful post-radiation mastitis, and one mastectomy with immediate reconstruction because of a poor cosmetic result. One cancer developed in the contralateral breast. Distant metastasis occurred in 14 women (10%), four of whom also had a local recurrence. The median time of local recurrence was 2.9 years (range 1.3–6.9 years) and that of distant metastasis 3.6 years (range 0.7–8.2 years) in the overall patient group.

**Table 2** Association of Tn-C in invasion border with other prognostic factors in axillary node-negative breast cancers

| | All tumours (%) | Tn-C-positive invasion border (%) | Chi-square $P$-value |
|---|---|---|---|
| All | 121[a] | 63 (52) | |
| DCIS + | 43 | 17 (40) | |
| DCIS – | 78 | 46 (59) | 0.04 |
| EIC + | 6 | 4 (67) | |
| EIC – | 115 | 59 (51) | 0.46 |
| erbB-2 + | 7 | 3 (43) | |
| erbB-2 – | 103 | 51 (50) | 0.73 |
| p53 + | 19 | 11 (58) | |
| p53 – | 92 | 47 (51) | 0.59 |
| Ki-67 + | 65 | 40 (62) | |
| Ki-67 – | 45 | 18 (40) | 0.03 |
| SPF high | 62 | 40 (65) | |
| SPF low | 51 | 19 (37) | 0.004 |
| Aneuploid | 44 | 23 (52) | |
| Diploid | 72 | 37 (51) | 0.93 |

[a]An invasion border was present in 121 tumours out of the 159 in the archival specimens available.

### Metastasis-free survival in the original patient group and prognostic factors

The 8-year metastasis-free survival (MFS) was 89% in the original patients. Age, histology, grade, erbB-2, p53, SPF and ploidy did not predict metastasis significantly in a univariate analysis. The MFS in tumours 13 mm or smaller was 94%, and 86% in those larger than 13 mm ($P$ = 0.01). The MFS in Ki-67-positive tumours was 84% and 96% in Ki-67-negative ones ($P$ = 0.04). When Tn-C was expressed in the invasion border the MFS was 84% compared with 98% when there was no such expression ($P$ = 0.03).

### Prognostic factors of distant and local recurrence in the overall patient group

In univariate analysis for metastasis, size ($P$ = 0.05), SPF of diploid tumours ($P$ = 0.0001) and Ki-67 ($P$ = 0.03) were significant prognostic factors, whereas grade ($P$ = 0.18), age ($P$ = 0.51), SPF of aneuploid tumours ($P$ = 0.77) or of all tumours as a group ($P$ = 0.73) and p53 ($P$ = 0.90) failed to show prognostic power as continuous variables. The prognostic comparison of the categorized variables is presented in Table 3. In addition, when the diploid tumours were dichotomized to low and high SPF, the relative risk for metastasis in the high group was 3.16 ($P$ = 0.05), whereas there was no such difference in the aneuploid tumours. Tn-C in the invasion border was statistically significant in predicting metastasis, and for local recurrence it was the only statistically significant prognostic factor (Table 3) with a hazard ratio of 11.0, CI 1.4–85.1, $P$ = 0.02.

The hazard ratio of metastasis in patients with a tumour larger than 13 mm was 2.6, CI 1.1–5.9, $P$ = 0.02, in those with Ki-67-positive tumours 5.5, CI 1.6–18.7, $P$ = 0.006, and in those with tumours with Tn-C expression in the invasion border 3.4, CI 1.1–10.4, $P$ = 0.03 (Table 3). When these three variables were introduced in a multivariate analysis, Ki-67 remained the only independent prognostic factor for metastasis (Table 4).

### DISCUSSION

Tn-C is produced by normal mesenchymal and epithelial cells as well as some carcinoma cells (Lightner et al, 1994; Ishihara et al, 1995). Tn-C produced by carcinoma cells has been suggested to facilitate the spreading of the carcinoma (Ishihara et al, 1995). Our recent observation suggests that the expression of Tn-C at the active site of epithelial–stromal invasion indicates a more aggressive disease (Jahkola et al, 1996). The present study shows that the expression of Tn-C at the site of invasion is correlated with a higher proliferation rate measured by flow cytometric analysis of SPF and the immunohistochemical detection of Ki-67 antigen, which gives further support to the active role of Tn-C in cancer dissemination.

Tumour size is a well-known risk factor in node-negative breast cancer for both distant and local recurrence. In a large study of

*© Cancer Research Campaign 1998*

**Table 3** Distributions of prognostic factors and hazard ratios (HRs) for local recurrence and metastasis in 158 women with 159 axillary node-negative breast cancers treated with breast-saving surgery and post-operative radiotherapy

| | Number of tumours | HR for local recurrence | P-value | HR for metastasis | P-value |
|---|---|---|---|---|---|
| Age <50 years | 56 | 1.0 | | 1.0 | |
| Age ≥50 years | 103 | 0.95 | 0.93 (NS) | 0.68 | 0.34 (NS) |
| Histology[a] | | | | | |
| Ductal | 95 | 1.0 | | 1.0 | |
| Lobular | 35 | 1.0 | | 1.0 | |
| Others | 29 | 0.34 | 0.30 (NS) | 0.37 | 0.18 (NS) |
| DCIS + | 57 | 1.0 | | 1.0 | |
| DCIS – | 102 | 0.77 | 0.63 (NS) | 1.07 | 0.87 (NS) |
| EIC + | 12 | 1.0 | | 1.0 | |
| EIC – | 147 | 1.04 | 0.97 (NS) | 0.97 | 0.96 (NS) |
| Grade[b] | | | | | |
| 1 | 51 | 1.0 | | 1.0 | |
| 2 | 34 | 1.0 | | 0.66 | |
| 3 | 26 | 2.86 | 0.30 (NS) | 1.72 | 0.35 (NS) |
| Size >13 mm | 66 | 1.0 | | 1.0 | |
| Size ≤13 mm | 91 | 0.80 | 0.69 (NS) | 0.39 | 0.02 |
| erbB-2 +[c] | 11 | | | 1.0 | |
| erbB-2 – | 131 | | – | 0.46 | 0.31 (NS) |
| p53 +[d] | 22 | 1.0 | | 1.0 | |
| p53 – | 121 | 0.88 | 0.87 (NS) | 0.58 | 0.29 (NS) |
| Ki-67 +[e] | 80 | 1.0 | | 1.0 | |
| Ki-67 – | 61 | 0.69 | 0.55 (NS) | 0.18 | 0.006 |
| SPF high[f] | 73 | 1.0 | | 1.0 | |
| SPF low | 72 | 1.37 | 0.58 (NS) | 0.47 | 0.10 (NS) |
| Aneuploid | 55 | 1.0 | | 1.0 | |
| Diploid | 95 | 1.82 | 0.37 (NS) | 1.75 | 0.24 (NS) |
| Expression of Tn-C in invasion border | | | | | |
| + | 63 | 1.0 | | 1.0 | |
| – | 58 | 0.09 | 0.02 | 0.30 | 0.03 |

[a]Others: two papillary, one medullary, two mucinous, 14 tubular, ten tubulolobular. For comparison with 'others' ductal and lobular carcinomas were combined. [b]Grade of 111 ductal and tubular carcinomas. [c]erbB-2 was stained only of the original patient material. Hazard ratio could not be computed because of no local recurrences in the erbB-2 positive group. [d]p53 cut-off 20% of nuclei positive. [e]Ki-67 cut-off 5% of nuclei positive. [f]SPF cut-off: median SPF of diploid tumours 2.4% and median of aneuploid tumours 8.4%.

**Table 4** Multivariate analysis (Cox proportional hazard model) of the three covariates statistically significant in univariate analysis of metastasis

| Covariate | P | HR | CI (95%) |
|---|---|---|---|
| Ki-67 (±, 5% cut off) | 0.03 | 10.0 | 1.3–77.2 |
| Tumour size (continuous) | 0.17 | 2.1 | 0.7–6.0 |
| Tn-C in invasion border | 0.34 | 1.8 | 0.56–5.8 |

1800 patients who received locoregional therapy and had an 8-year follow-up, tumour size was the strongest predictor for distant recurrence. A high proliferation activity measured by the [$^3$H]-thymidine labelling index provided additional prognostic information in intermediate size (1–2 cm) tumours. Cell proliferation was the most significant predictor for locoregional relapse (Silvestrini et al, 1995). As in our study, SPF has been found to be a significant prognostic factor of distant metastasis in diploid but not in aneuploid tumours (Clark et al, 1989). A similar observation has been made in soft-tissue sarcomas (Huuhtanen et al, 1996). The prognostic value of immunohistochemically detected erbB-2 and p53 protein in early node-negative breast cancer is controversial (Silvestrini et al, 1993; Ravdin and Chamness, 1995; Rosen et al, 1995). In this study those markers did not show prognostic power for either local or distant recurrence.

Young age, tumour size, presence of carcinoma at the resection margin and the presence of an extensive intraductal component (EIC) are associated with a higher risk of local recurrence after breast-conserving surgery (Boyages et al, 1990; Holland et al, 1990; Borger et al, 1994; Gage et al, 1996; Schnitt et al, 1987). In the present study EIC was present in only 8% of the tumours compared with 20% in many of the previous studies, probably because the selection criteria for breast conservation had been strict: only tumours up to 2 cm in diameter (measured clinically) and with histologically clear margins were accepted. In this selected material the expression of Tn-C in the invasion border was the only marker that predicted local recurrence. Our results suggest that patients with carcinomas that do not express Tn-C in the invasion border do not

© Cancer Research Campaign 1998

need adjuvant treatments. On the other hand, patients with tumours expressing Tn-C in the area of invasion have an increased risk of developing both local and distant recurrences.

Small tumour size, low grade and old age have been proposed as selection criteria for patients who might not benefit from radiotherapy (Clark et al, 1992; Veronesi et al, 1993; Liljegren et al, 1994). However, in a highly selected group of 87 patients treated with breast-conserving surgery without radiotherapy, the rate of local recurrence rose to 16% when the median follow-up time was 56 months, therefore this prospective clinical trial was closed (Schnitt et al, 1996). Because of the lack of reliable exclusion criteria the importance of post-operative radiotherapy for all patients has been reappraised (Morrow et al, 1995). The expression of Tn-C in the invasion border seems to be a biological marker of a higher risk for local relapse, and the absence of Tn-C might be used as an adjunct to define a group that could be left without radiotherapy.

Our results suggest that more radical surgery should be considered, adjuvant treatment recommended and that follow-ups should be more frequent and continued longer when Tn-C is expressed in the tumour invasion border. Additional studies are needed to establish whether Tn-C expression in the invasion border can be used as a criterion to select conservatively treated axillary node-negative breast cancer patients for adjuvant chemotherapy or hormonal therapy to reduce the risk of both local and distant disease spread.

### ACKNOWLEDGEMENTS

The skilful technical assistance of Ms L Pirjamo, A Takkinen, M-L Piironen, M Shoultz, P Peltokangas, A Mäki and Mr R Karppinen is acknowledged. Professor S Sarna has given valuable suggestions concerning the statistical presentation.

### REFERENCES

Bloom HJG and Richardson WW (1957) Histological grading and prognosis in breast cancer. A study of 1409 cases of which 359 have been followed for 15 years. *Br J Cancer* **11**: 359–377

Borger J, Kemperman H, Hart A, Peterse H, van Dongen J and Bartelink H (1994) Risk factors in breast-conservation therapy. *J Clin Oncol* **12**: 653–660

Boyages J, Recht A, Connolly JL, Schnitt SJ, Gelman R, Kooy H, Love S, Osteen RT, Cady B, Silver B and Harris JR (1990) Early breast cancer: predictors of breast recurrence for patients treated with conservative surgery and radiation therapy. *Radiother Oncol* **19**: 29–41

Clark GM, Dressler LG, Owens MA, Pounds G, Oldaker T and McGuire WL (1989) Prediction of relapse or survival in patients with node-negative breast cancer by DNA flow cytometry. *N Engl J Med* **320**: 627–633

Clark RM, McCulloch PB, Levine MN, Lipa M, Wilkinson RH, Mahoney LJ, Basrur VR, Nair BD, McDermot RS, Wong CS and Corbett PJ (1992) Randomized clinical trial to assess the effectiveness of breast irradiation following lumpectomy and axillary dissection for node-negative breast cancer. *J Natl Cancer Inst* **84**: 683–689

EBCTCG (1992) Systemic treatment of early breast cancer by hormonal, cytotoxic, or immune therapy. 133 randomised trials involving 31,000 recurrences and 24,000 deaths among 75,000 women. Early Breast Cancer Trialists' Collaborative Group [see comments]. *Lancet* **339**: 1–15 and 71–85

EBCTCG (1995) Effects of radiotherapy and surgery in early breast cancer. *N Engl J Med* **333**: 1444–1455

Ferguson JE, Schor AM, Howell A and Ferguson MW (1990) Tn-C distribution in the normal human breast is altered during the menstrual cycle and in carcinoma. *Differentiation* **42**: 199–207

Fisher B, Anderson S, Redmond CK, Wolmark N, Wickerham DL and Cronin WM (1995) Renalysis and results after 12 years of follow-up in a randomized clinical trial comparing total mastectomy with lumpectomy with or without irradiation in the treatment of breast cancer. *N Engl J Med* **333**: 1456–1461

Fisher ER, Costantino J, Fisher B, Redmond C and CNSABaBP Investigators (1993). Pathologic findings from the National Surgical Adjuvant Breast Project (Protocol 4) – Discriminants for 15-year survival. *Cancer* **71**: 2141–2150

Gage I, Schnitt SJ, Nixon AJ, Silver B, Recht A, Troyan SL, Eberlein T, Love SM, Gelman R, Harris JR and Connolly JL (1996) Pathologic margin involvement and the risk of recurrence in patients treated with breast-conserving therapy. *Cancer* **78**: 1921–1928

Gelber RD and Goldhirsch A (1994) Radiotherapy to the conserved breast: is it avoidable if the cancer is small? *J Natl Cancer Inst* **86**: 652–654

Haffty BG, Fisher D, Rose M, Beinfield M and McKhann C (1991) Prognostic factors for local recurrence in the conservatively treated breast cancer patient: a cautious interpretation of the data. *J Clin Oncol* **9**: 997–1003

Hedley DW, Friedlander ML, Taylor IW, Rugg CA and Musgrove EA (1983) Method for analysis of cellular DNA content of paraffin-embedded pathological material using flow cytometry. *J Histochem Cytochem* **31**: 1333–1335

Holland R, Connolly JL, Gelman R, Mravunac M, Hendriks JHCL, Verbeek ALM, Schnitt SJ, Silver B, Boyages J and Harris JR (1990) The presence of an extensive intraductal component following a limited excision correlates with prominent residual disease in the remainder of the breast. *J Clin Oncol* **8**: 113–118

Howeedy AA, Virtanen I, Laitinen L, Gould NS, Koukoulis GK and Gould VE (1990) Differential distribution of tenascin in the normal, hyperplastic, and neoplastic breast. *Lab Invest* **63**: 798–806

Huuhtanen R, Wiklund T, Blomqvist C, Virolainen M, Yi P and Tribukait B (1996) High S-phase fraction is an adverse prognostic sign in diploid soft-tissue sarcomas. *Cancer* **77**: 1815–1822

Ishihara A, Yoshida T, Tamaki H and Sakakura T (1995) Tenascin expression in cancer cells and stroma of human breast cancer and its prognostic significance. *Clin Cancer Res* **1**: 1035–1041

Jahkola T, Toivonen T, Von Smitten K, Blomqvist C and Virtanen I (1996) Expression of tenascin in invasion border of early breast cancer correlates with higher risk of distant metastasis. *Int J Cancer (Pred Oncol)* **69**: 445–447

Jones PL, Cowan KN and Rabinovitch M (1997) Tenascin-C, proliferation and subendothelial fibronectin in progressive pulmonary vascular disease. *Am J Pathol* **150**: 1349–1360

Lightner VA, Marks JR and McCachren SS (1994) Epithelial cells are an important source of tenascin in normal and malignant human breast tissue. *Exp Cell Res* **210**: 177–184

Liljegren G, Holmberg L, Adami H-O, Westman G, Graffman S and Bergh J (1994) Sector resection with or without postoperative radiotherapy for stage I breast cancer. Five year results of a randomized trial. *J Natl Cancer Inst* **86**: 717–722

Mansour EG, Ravdin PM and Dressler L (1994) Prognostic factors in early breast carcinoma. *Cancer* **74**: 381–400

Morrow M, Harris JR and Schnitt SJ (1995) Local control following breast-conserving surgery for invasive cancer: results of clinical trials. *J Natl Cancer Inst* **87**: 1669–1673

Ravdin PM and Chamness GC (1995) The c-erbB-2 proto-oncogene as a prognostic and predictive marker in breast cancer: a paradigm for the development of other macromolecular markers – a review. *Gene* **159**: 19–27

Rosen PP, Lesser ML, Arroyo CD, Cranor M, Borgen P and Norton L (1995) p53 in node-negative breast carcinoma: an immunohistochemical study of epidemiologic risk factors, histologic features, and prognosis. *J Clin Oncol* **13**: 821–830

Sakakura T, Ishihara A and Yatani R (1991) Tn-C in mammary gland development: from embryogenesis to carcinogenesis. In *Regulatory Mechanisms in Breast Cancer*, Lippman ME (ed.) pp. 383–400. Advances in Cellular and Molecular Biology of Breast Cancer. Kluwer Academic Publishers: Boston

Schnitt SJ, Connolly JL, Khettry U, Mazoujian G, Brenner M, Silver B, Recht A, Beadle G and Harris JR (1987) Pathologic findings on re-excision of the primary site in breast cancer patients considered for treatment by primary radiation therapy. *Cancer* **59**: 675–681

Schnitt SJ, Hayman J, Gelman R, Eberlein TJ, Love SM, Mayzel K, Osteen, RT, Nixon AJ, Pierce S, Connolly JL, Cohen P, Schneider L, Silver, B, Recht A and Harris JR (1996) A prospective study of conservative surgery alone in the treatment of selected patients with stage I breast cancer. *Cancer* **77**: 1094–1100

Silvestrini R, Benini E, Daidone MG, Veneroni S, Boracchi P, Cappelletti V, Fronzo GD and Veronesi U (1993) p53 as an independent prognostic marker in lymph node-negative breast cancer patients. *J Natl Cancer Inst* **85**: 965–970

Silvestrini R, Daidone MG, Luisi A, Boracchi P, Mezzetti M, Fronzo GD, Andreola S, Salvadori B and Veronesi U (1995) Biologic and clinicopathological factors as indicators of specific relapse types in node-negative breast cancer. *J Clin Oncol* **13**: 697–704

 © Cancer Research Campaign 1998

Tiitta O, Wahlström T, Paavonen J, Linnala A, Sharma S, Gould VE and Virtanen I (1992) Enhanced tenascin expression in cervical and vulvar koilocytotic lesions. *Am J Pathol* **141**: 907–913

Veronesi U, Luini A, Vecchio MD, Greco M, Galimberti V, Merson M, Rilke R, Sacchini V, Saccozzi R, Savio T, Zucali R, Zurrida S and Salvadori B (1993) Radiotherapy after breast-preserving surgery in women with localized cancer of the breast. *N Engl J Med* **328**: 1587–1591

Veronesi U, Marubini E, Vecchio MD, Manzari A, Andreola S, Greco M, Luini A, Merson M, Saccozzi R, Rilke F and Salvadori B (1995) Local recurrences and distant metastasis after conservative breast cancer treatments: partly independent events. *J Natl Cancer Inst* **87**: 19–27

Victorzon M, Roberts PJ, Haglund C, von Boguslawski K and Nordling S (1996) Ki-67 immunoreactivity, ploidy and S-phase fraction as prognostic factors in patients with gastric carcinoma. *Oncology* **53**: 182–191

Whelan T, Clark R, Roberts R, Levine M, Foster G and IotOCO Group (1994) Ipsilateral breast tumour recurrence postlumpectomy is predictive of subsequent mortality: results from a randomized trial. *Int J Radiat Oncol* **30**: 11–6

Yoshida T, Ishihara A, Hirokawa Y, Kusakabe M and Sakakura T (1995) Tenascin in breast cancer development – is epithelial tenascin a marker for poor prognosis. *Cancer Lett* **90**: 65–73

© Cancer Research Campaign 1998